# Semantic Processing in Bilingual Aphasia: Evidence of Language Dependency

**DOI:** 10.3389/fnhum.2019.00205

**Published:** 2019-06-14

**Authors:** Marco Calabria, Nicholas Grunden, Mariona Serra, Carmen García-Sánchez, Albert Costa

**Affiliations:** ^1^Center for Brain and Cognition, Pompeu Fabra University, Barcelona, Spain; ^2^Hospital de la Santa Creu i Sant Pau, Barcelona, Spain; ^3^Institució Catalana de Recerca i Estudis Avançats, Barcelona, Spain

**Keywords:** bilingual aphasia, semantic control, cycling naming, language dependency, executive control, language control

## Abstract

Individuals with aphasia frequently show lexical retrieval deficits due to increased interference of semantically related competitors, a phenomenon that can be observed in tasks such as naming pictures grouped by semantic category. These deficits are explained in terms of impaired semantic control, a set of abilities that are to some extent dependent upon executive control (EC). However, the extent to which semantic control abilities can be affected in a second and non-dominant language has not been extensively explored. Additionally, findings in healthy individuals are inconclusive regarding the degree to which semantic processing is shared between languages. In this study, we explored the effect of brain damage on semantic processing by comparing the performance of bilingual individuals with aphasia on tasks involving semantic control during word production and comprehension. Furthermore, we explored whether semantic deficits are related to domain-general EC deficits. First, we investigated the naming performance of Catalan–Spanish bilinguals with fluent aphasia and age-matched healthy controls on a semantically blocked cyclic naming task in each of their two languages (Catalan and Spanish). This task measured semantic interference in terms of the difference in naming latencies between pictures grouped by the same semantic category or different categories. Second, we explored whether lexical deficits extend to comprehension by testing participants in a word-picture matching task during a mixed language condition. Third, we used a conflict monitoring task to explore the presence of EC deficits in patients with aphasia. We found two main results. First, in both language tasks, bilingual patients’ performances were more affected than those of healthy controls when they performed the task in their non-dominant language. Second, there was a significant correlation between the speed of processing on the EC task and the magnitude of the semantic interference effect exclusively in the non-dominant language. Taken together, these results suggest that lexical retrieval may be selectively impaired in bilinguals within those conditions where semantic competition is higher, i.e.,- in their non-dominant language; this could possibly be explained by an excessive amount of inhibition placed upon this language. Moreover, lexico-semantic impairments seem to be at least somewhat related to conflict monitoring deficits, suggesting a certain degree of overlap between EC and semantic control.

## Introduction

Lexical retrieval deficits in aphasia have many different potential sources of impairment including dysfunction in semantic selection, lexical selection, and/or phonological processing (Laine and Martin, 2006). Some more recent views, specifically those that take into account connectionist models, have broadly defined two main levels of retrieval: the first stage comprised of meaning and grammar and the second of phonological structure and content (Schwartz, 2014). In the present study, we aim to investigate the role of semantic control, defined as one of the mechanisms within the semantic network, in lexical retrieval deficits within patients with bilingual aphasia.

Semantic control can be defined as a set of processes that enable an individual to modulate retrieval of information based on the contextual cognitive demand (Lambon Ralph et al., 2017) and can be distinguished from semantic representation within the semantic cognition network (Jefferies, 2013; Lambon Ralph et al., 2017). To some extent, this idea coincides with the concept of ‘access deficits’ in semantic aphasia as opposed to the ‘storage deficits’ in semantic dementia (for a review, see Mirman and Britt, 2004). Of particular interest within the context of post-stroke aphasia is the control element of this semantic framework, since semantic memory is usually spared (Jefferies and Lambon Ralph, 2006; Jefferies et al., 2008; Rogers et al., 2015).

Findings from neuroimaging studies have distinguished a semantic control network that includes the left prefrontal and temporo-parietal cortices, as opposed to the anterior temporal lobes, serving as crucial elements for conceptual representations (Noonan et al., 2010, 2013). Interestingly, the neural model of semantic cognition proposed by Lambon Ralph et al. (2017) shows an overlap with the bilingual language control network in which prefrontal areas are engaged in conflict resolution and the posterior areas (inferior parietal lobules) in language selection (Abutalebi and Green, 2016; Calabria et al., 2018). Most studies agree that a second language (L2) is mainly acquired through the same neural devices responsible for the first language (L1) and that the brain systems associated with the linguistic processing are shared between the two languages (e.g., Perani and Abutalebi, 2005; Abutalebi and Green, 2007). Broadly speaking, we did not expect there to be a difference in semantic control abilities for L1 and L2. However, some differences have been reported between the two languages when bilinguals have to process semantic incongruence. In their review, Moreno et al. (2008) concluded that semantic processing in L2 is delayed, as measured by a delayed peak latency of the event-related potential (N400) associated with semantic violation, thus suggesting differences in semantic integration between the bilinguals’ two languages. Similarly, some bilingual models of speech production claimed that lexico-semantic representation might function differently for a bilingual’s two languages (Kroll and Stewart, 1994; Gollan et al., 2008; Kroll et al., 2015; for a review see Branzi et al., 2018).

In the present study, we wanted to test the hypothesis of language-independency of semantic control by investigating the performance of bilingual patients with aphasia on semantic control tasks in their two languages (Catalan and Spanish). To do so, we used the semantic blocked cycling naming task that has been extensively used to investigate semantic interference both in healthy individuals (Damian and Bowers, 2003; Belke et al., 2005; Damian and Als, 2005; Navarrete et al., 2012; Belke, 2017) and in monolingual patients with aphasia (McCarthy and Kartsounis, 2000; Wilshire and McCarthy, 2002; Schnur et al., 2006; Biegler et al., 2008; Harvey and Schnur, 2015) as a measurement of semantic competition during lexical selection.

### Semantic Processing in Healthy Bilinguals: Language-Dependent or Independent?

The results of a series of behavioral and neuroimaging studies agree with the hypothesis that there are similar principles of semantic representation across languages. For instance, studies that used semantic cross-language priming found that, with highly proficient bilinguals, the magnitude of word priming between semantically related words is similar irrespective of the language direction (e.g., Zeelenberg and Pecher, 2003; Perea et al., 2008; Travis et al., 2017). Furthermore, when bilinguals have to name pictures in a semantically demanding task, they show a similar magnitude of semantic interference in both L1 and L2, suggesting that semantic control abilities are independent of the language being utilized (Runnqvist et al., 2012).

Despite that some qualitative differences between languages have been found, the main results of relevant studies support the hypothesis of a shared conceptual/semantic system across languages (Francis, 1999, 2005), as proposed in some models of bilingual production and comprehension (BIA+ model: Dijkstra and van Heuven, 2003; ICM: Green, 1986; RHM: Kroll and Stewart, 1994).

A second line of research has investigated the underlying neural network of semantic processing in bilinguals on a variety of semantic tasks. Some studies concur that bilinguals show similar activation while they are processing semantic representations in their L1 and L2, identifying a language-invariant semantic network that includes the inferior temporal lobe (Grogan et al., 2009), superior temporal lobes (Chee et al., 2001; Pillai et al., 2003), frontal (Illes et al., 1999; Chee et al., 2001) or a more widely distributed set of language areas (Correia et al., 2014; Van de Putte et al., 2017). One exception is a study conducted by Klein et al. (2006) that found activation in the putamen when subjects performed L1-L2 translation but not the inverse direction that coincided with an otherwise complete overlap of activation for the two languages during a word generation task.

Finally, some evidence of the possible language-dependency of semantic processing comes from sentence processing in bilinguals. Specifically, the event-related potential component (N400) that indexes semantic violation has been found to be consistently delayed in its peak latency for L2 relative to L1 (for a review see Moreno et al., 2008).

Therefore, although most studies agree that bilinguals show shared semantic networks for L1 and L2, some research revealed the presence of language-dependent processes, possibly related to the type of task used to assess semantic representation or control. These results are in line with what some other models of bilingual speech production have proposed. For instance, the Revised Hierarchical Model (RHM) by Kroll and Stewart (1994) assumes that the L1 lexicon is larger than that of L2 and that the connections between L1 concepts are stronger than those between L2, which are thought to be attached to the L1 lexicon. Similarly, the ICM model by Green (1986) would predict different degrees of inhibitory control in each language that, once applied at the schema level, would modulate lexical selection according the dominance of the two languages.

### Semantic Deficits in Bilingual Speakers With Brain Damage

Research that has investigated semantic deficits in bilingual patients with neurodegenerative disorders has shown similar impairments across languages, suggesting that semantic processing is language-independent (Mendez et al., 1999; Hernández et al., 2008, 2010). In the first study by Hernández et al. (2008), patient JPG had similar category-specific deficits in both languages (Spanish and Catalan) with worse performance in naming verbs than nouns. In a further study, Hernández et al. (2010) found that the semantic memory deficits of JFF (a Catalan–Spanish bilingual patient) had a similar influence on his performance while he performed word translation in both language directions. In both studies, only some qualitative differences of errors between languages were reported, but the main result supports a shared conceptual representation across languages (Francis, 1999, 2005).

Also, studies performed on bilingual patients with aphasia have found that the representational level of knowledge is shared between languages. For example, Siyambalapitiya et al. (2013) found that their patient (SN) not only showed intact semantic priming in both languages, but also in the cross-language condition (from English to Spanish), supporting a language-independent nature of bilingual semantic memory. Other research within bilingual aphasia has uncovered a more complex picture that would support the notion that, in post-stroke aphasia, patients’ deficits arose from dysfunction in the control part of the semantic system instead of representational system of knowledge as in dementia patients [see the controlled semantic cognition (CSC) model by Lambon Ralph et al., 2017]. Some of this data comes from the study of cross-language generalization using semantic-based training (for a more extended discussion on cross-language issues in aphasia see Faroqi-Shah et al., 2010; Khachatryan et al., 2016). For instance, Kiran and Roberts (2010) found that the only one of the four patients they tested after semantic-based naming treatment improved in the untrained language, suggesting that providing semantic information to improve lexical retrieval has little to no cross-language transfer. Kiran et al. (2013b) in a further study found a similar result of limited cross-language generalization for semantic representations. Interestingly, they proposed that the degree of cross-language transfer might be explained by the integrity of two independent mechanisms: the first being a generalized mechanism involved in the spreading of activation brought about via treatment and the second being inhibitory control which, in the case of bilingual speakers, would interfere with the activation level of their two languages (Green, 1986). Therefore, the degree of within- and between-language generalization depends on the interplay of these two mechanisms, namely how inhibitory control works to allow semantic activation to increase in one language and/or in both.

Interestingly, the idea that EC plays a role during semantic processing is similar to what was proposed by Lambon Ralph et al. (2017) for monolinguals. These authors claim that, along with an amodal ‘hub’ which functions by integrating different sources of information (Patterson et al., 2007), there are EC mechanisms that supervise how activation spreads throughout the semantic representation network. That is, there exists a combination of two systems: one representational (temporo-parietal) and one for control (frontally distributed), with the latter being more closely related to semantic control deficits in monolingual patients with aphasia (Harvey and Schnur, 2015).

Therefore, following the idea of the CSC model, we aimed to investigate whether semantic control may be differentially affected in the two languages of bilinguals post-stroke. To do so, we employed a blocked naming task that allowed us to manipulate the amount of interference during word retrieval for semantically-related competitors. This type of paradigm has been extensively used in studies with monolingual patients with aphasia to test the root causes of word retrieval deficits (e.g., Wilshire and McCarthy, 2002; Biegler et al., 2008; Schnur et al., 2009; Scott and Wilshire, 2010; Harvey and Schnur, 2015). According to some authors, this task can also help test whether word retrieval deficits can be explained in terms of an increased excitation or an excessive inhibition applied to semantic competitors, resulting in the target words being less available during lexical selection (Schnur et al., 2006).

Moreover, to specifically test the relationship between semantic control processes and EC, we tested patients on a conflict monitoring task. The inclusion of this task was motivated by a new body of research with bilingual aphasic patients that highlights the cross-talk between deficits in language control and in domain-general EC (Dash and Kar, 2014; Gray and Kiran, 2015; Faroqi-Shah et al., 2016; for a review on this issue see Calabria et al., 2018).

### The Present Study

To investigate semantic control during speech production in patients, we employed a semantic blocked cyclic naming task. In this paradigm, participants were required to name pictures in two conditions: (a) homogeneous, where pictures belonged to the same semantic category (e.g., only animals), and (b) heterogeneous, where pictures belonged to different semantic categories (e.g., animals, furniture, tools, etc.). The latencies in the naming of elements in the heterogeneous condition become faster over repetitions (cycles) whereas those in the homogeneous generally remain constant after the second cycle (e.g., Damian and Bowers, 2003; Belke et al., 2005; Damian and Als, 2005; Navarrete et al., 2012; Crowther and Martin, 2014; Belke, 2017). The difference in naming latencies between these two conditions is an index of semantic interference that is increased in patients with aphasia compared to healthy individuals (e.g., Schnur et al., 2006, 2009; Biegler et al., 2008; Scott and Wilshire, 2010) due to hyper-activation or excessive inhibition of semantic competitors brought on by their language impairments. This agrees with the view that lexical selection is a competitive process (for a recent review see Nozari and Hepner, 2018). The automatic activation of semantically related items spreads to their corresponding lexical representations and the target word competes for selection (for non-competitive models see Costa et al., 1999; Mahon et al., 2007).

The general hypothesis about semantic control in bilingualism was that if semantic control was language-independent, we expected to see a similar increase of semantic interference in both languages in patients with aphasia compared to healthy controls. Indeed, according to the models that have proposed that lexical selection in bilinguals is qualitatively similar to that of monolinguals, we should expect language-independency of semantic control (Costa et al., 1999; Caramazza and Costa, 2000; La Heij, 2005; Finkbeiner et al., 2006).

On the other hand, if semantic control was language-dependent, we expected to see higher interference in one language compared to the other. Presumably, more semantic impairment would occur in the non-dominant language if it were related to EC deficits (e.g., Abutalebi and Green, 2007, 2016) or had weaker connections between lexical and semantic units (e.g., Kroll and Stewart, 1994).

In order to assess the integrity of semantic representations, we employed a bilingual word-picture matching task. Participants were required to match a picture presented on the screen with one of two word options (semantically related, same category). The main reasoning behind the inclusion of this task was to measure the accuracy of patients as compared to healthy controls on the task and thus to exclude the possibility of any representational deficits in semantic memory. We adopted a bilingual version of the matching task because this type of paradigm allowed us to test both languages in the same task and because we have already seen previous evidence that it serves as a robust task for testing comprehension in bilinguals (Macizo et al., 2010; Mosca and de Bot, 2017).

Additionally, we investigated the integrity of EC with a conflict monitoring task in patients and healthy controls. This task has been used previously in studies with bilingual patients with the aim to investigate the relationship between language control and EC deficits (Green et al., 2010; Gray and Kiran, 2015). We correlated patients’ performance on this EC task with the semantic blocked cyclic naming task, with the degree of the correlation indicating to what extent the two domains of control overlap. The available literature on this issue reports mixed findings and the number of studies performed with bilingual patients after stroke in which the two domains have been compared is very slim (for a review see Calabria et al., 2018), resulting in a need for further research on this issue. Therefore, an overlap of deficits in both domains would suggest that domain-general EC is also involved in language selection. That is, the hyper-activation or -inhibition upon the semantic competitor during lexical selection would be intimately related to non-linguistic EC processes (inhibitory control and/or conflict resolution), as predicted by the ‘executive selection account’ (Wilshire and McCarthy, 2002).

To summarize, the current study was undertaken to explore two issues in the context of semantic control and bilingualism:

(1) Are semantic control processes language-dependent and differentially affected by brain damage in a bilingual’s two languages?
(2) Are semantic control processes dependent on general-domain EC mechanisms such as conflict monitoring and resolution?

## Materials and Methods

### Participants

A total of 11 Catalan–Spanish patients with bilingual aphasia were recruited from the Speech Therapy Unit of Hospital de la Santa Creu i Sant Pau in Barcelona. All patients were speakers of both Catalan and Spanish prior to stroke, exhibited adequate hearing and vision, demonstrated stable health status and were in the chronic stage for language disorders (more than 1 year post-injury). The etiology was brain tumor for one patient (Pt2) and cerebrovascular (either ischemic or hemorrhagic stroke) for all other patients. All patients had lesions localized in the left hemisphere.

A group of 13 healthy individuals also participated in the study as controls; their demographic and linguistic characteristics were matched to those of patients with aphasia.

#### Language Assessment

To define the type and the degree of language impairment, the Spanish version of the Western Aphasia Battery (Kertesz and Pascual-Leone García, 1990) was administered by Dr. García Sánchez, a clinical neuropsychologist with expertise in aphasia from the same hospital. The WAB is a comprehensive test of language functions with a relatively short test administration time (30–60 min) and includes four language subtests which assess spontaneous speech, comprehension, repetition, and naming to calculate an Aphasia Quotient (AQ). Patients were only tested in Spanish since a Catalan version of the WAB is not currently available.

According to WAB assessment, one patient was classified as having conduction aphasia, two with Wernicke’s aphasia and eight classified as presenting anomic aphasia. The degree of language impairment ranged from mild to moderate (55.6 to 84.5 out of 100) and the mean values for each subtest were: 14.1/20 (±2.6) for Fluency, 8.2/10 (±1.2) for Comprehension; 7.4/10 (±1.7) for Repetition, and 7.4/10 (±1.1) for Naming (see Table 1 for details).

**Table 1:** Individual scores of patients for the Western Aphasia Battery and Bilingual Aphasia Test (Part C).

Patients	Aphasia type	Months post-onset	Aphasia quotient^∗^ (max. 100)	Severity	Fluency (max. 20)	Comprehension (max. 10)	Repetition (max. 10)	Naming (max. 10)	DL	NDL	BAT-C DL (max. 48)	BAT -C NDL (max. 48)	BAT-C-DL vs. NDL *p*-values	LogType/LogToken DL	LogType/LogToken NDL
Pt1	ANOMIC	73	71.00	Moderate	12.00	8.50	8.10	8.10	CAST	CAT	29	24	0.405	0,92	0,92
Pt2	CONDUCTION	131	84.50	Mild	18.00	9.25	6.40	8.60	CAT	CAST	24	19	0.407	0,87	0,88
Pt3	ANOMIC	122	64.00	Moderate	10.00	7.30	8.20	6.50	CAT	CAST	21	18	0.675	0,85	0,88
Pt4	ANOMIC	46	73.40	Moderate	15.00	8.40	7.00	6.30	CAT	CAST	29	26	0.675	0,86	0,87
Pt5	WERNICKE	108	75.70	Mild to moderate	16.00	6.75	6.20	8.90	CAT	CAST	28	24	0.533	0,83	0,89
Pt6	ANOMIC	49	79.90	Mild to moderate	14.00	9.55	8.60	7.30	CAST	CAT	14	12	0.817	0,91	0,91
Pt7	ANOMIC	21	88.20	Mild	15.00	9.50	9.70	8.90	CAT	CAST	30	30	0.830	0,86	0,83
Pt8	ANOMIC	119	57.60	Moderate	10.00	6.90	7.10	5.80	CAT	CAST	16	17	0.828	0,80	0,80
Pt9	WERNICKE	84	56.30	Moderate	13.00	6.25	3.20	5.70	CAT	CAST	17	9	0.106	0,79	0,77
Pt10	ANOMIC	66	92.40	Mild	18.00	10.00	9.60	8.10	CAST	CAT	46	36	*0.007*	0,95	0,89
Pt11	ANOMIC	14	72.20	Moderate	14.00	7.90	7.30	6.90	CAT	CAST	26	28	0.834	0,87	0,88

*Aphasia Quotient (AQ). Range of severity of the scores: 0–25: Very severe; 26–50: Severe; 51–75: Moderate: > 76: Mild. DL: dominant language; NDL: non-dominant language.*

Patients’ language abilities were also tested using part C of the Bilingual Aphasia Test (BAT, Paradis and Libben, 1987) which assesses cross-language abilities over four subtests: Word Recognition, Word Translation, Sentence Translation, and Grammatical Judgment. In Word Recognition, patients were asked to select the correct translation for each word from a list of 10 possible choices (5 words per language; max. score = 10). In the Word Translation task, patients needed to verbally supply the translation of a word spoken by the examiner (10 words per language; max. score = 20). Increasing in difficulty, subjects then were asked in the Sentence Translation task to provide a translation of a sentence that could be repeated a maximum of three times by the examiner (scoring based on correct translations of 3 sections of each sentence for 6 sentences in each language; max. score = 36). Finally, in Grammatical Judgment, patients were asked to determine whether a sentence spoken by the examiner was grammatically correct and, if incorrect, how to fix it (scoring based on correct judgment of grammatical structure and accurate correction of grammatical mistakes if applicable for 8 sentences per language; max. score = 28). These subtests of the BAT-C were administered by a bilingual neuropsychologist, completing all four tasks in one direction of translation followed by the same four tasks in the other direction (i.e., Catalan to Spanish in all tasks followed by Spanish to Catalan).

Furthermore, to have an additional measure of language impairment in their two languages, we asked patients to describe two complex picture scenes: the Cookie Theft Picture (Goodglass and Kaplan, 1972) and the scene description from the WAB. They were instructed to use Catalan to describe the scene in one session and Spanish in the other, with this order counterbalanced across subjects. If some features of the pictures were neglected, the experimenter pointed to them and asked the patient to mention them. Speech was recorded and subsequently analyzed off-line. We collected one recording for each language, each lasting 3 min. After transcribing the descriptions in each language, the total number of words (tokens) and the number of different words (type) were calculated. In order to reduce the impact of sample size, we calculated the individual token-type ratio for each language by using the following transformation log_type_/log_token_ (Kong, 2016).

#### Language Profile

Language history and dominance were determined by means of a questionnaire administered to the participants and an interview with them. Pre-morbid language proficiency in the two languages (Catalan and Spanish) was self-rated by each participant on a four-point scale of their abilities of speaking, comprehension, writing and reading (1 = poor, 2 = regular, 3 = good, 4 = perfect). As can be appreciated in Table 2, both patients and healthy controls were highly proficient in all four linguistic domains (see also Appendix I for individual data). Moreover, participants were considered early bilinguals as, on average, they were first regularly exposed to their non-dominant language at 6 years of age, thus not differing significantly from the exposure to their dominant language. Finally, language usage was rated based on ten questions in which participants were required to report with what frequency they spoke each of the two languages across different periods of their lives. The final score was transformed into a percentage (from 0 meaning using only Spanish to 100% meaning using only Catalan, around 50% translating to balanced use of the two languages). Both patients and healthy controls reported equal amounts of Catalan and Spanish usage and thus would be considered balanced bilinguals.

**Table 2:** Socio-demographic and linguistic characteristics of the samples.

	Patients with aphasia		Healthy controls		
	(*n* = 11)		(*n* = 13)		
	*M*	*SD*	*M*	*SD*	*p*-values
Age (years)	58.2	6.4	55.4	4.1	*0.29*
Education (years)	13.6	1.7	14.4	1.2	*0.18*
Age of regular dominant language use	2.1	0.7	2.2	0.3	*0.52*
Age of regular non-dominant language use	4.6	1.6	4.8	1.2	*0.75*
Language proficiency (1–4)					
*Dominant language*					
Speaking	4.0	0.0	3.9	0.3	*0.35*
Comprehension	4.0	0.0	4.0	0.0	*–*
Reading	3.7	0.6	4.0	0.0	*0.21*
Writing	3.7	0.6	3.9	0.3	*0.21*
*Non-dominant language*	3.9	0.3	4.0	0.0	*0.30*
Speaking					
Comprehension	3.9	0.3	4.0	0.0	*0.30*
Reading	3.9	0.4	3.8	0.4	*0.51*
Writing	3.7	0.6	4.0	0.0	*0.16*
% Language use	54.5	15.3	45.1	20.8	*0.22*

The bilinguals that participated in this study acquired their two languages at the same time and it is difficult to say which would be their L1 or L2. Therefore, we used the terms ‘dominant’ and ‘non-dominant’ instead of L1 and L2 to refer to their languages. The use of ‘dominant’ refers to the language that they prefer to use (or they feel more comfortable speaking), even if they reported that their ‘non-dominant’ language was at the same level of proficiency and frequency of usage as their dominant. According to this definition, 3 patients and 3 healthy controls were classified as Spanish-dominant bilinguals while the rest were classified as Catalan-dominant bilinguals.

### Materials and Procedure

The experimental software used for the administration of all tasks was DMDX (Forster and Forster, 2003). All the participants performed three experimental tasks: the semantic blocked cyclic naming task, the bilingual word-picture matching task, and the flanker task. Before starting the experimental procedure, the patients signed an informed consent approved by the ‘Parc de Salut MAR’ Research Ethics Committee under the reference number: 2018/8029/I.

#### Semantic Blocked Cyclic Naming Task

Stimuli consisted of 32 pictures total with 8 exemplars from 4 semantic categories (animals, vegetables, kitchen tools, and furniture) selected from the Snodgrass and Vanderwart (1980) database (see Appendix II for the details of the stimulus). Participants were required to name 8 blocks of pictures: 4 blocks containing semantically related items (Homogenous) and 4 blocks containing semantically unrelated items (Heterogeneous). For some participants, two Homogenous blocks were followed by four Heterogeneous and then two Homogenous blocks whereas, for others, this pattern was reversed. Sets of 16 different pictures for each language were presented four times (cycles) in 4 Homogenous as well as 4 Heterogeneous blocks, with a total number of 128 naming trials per participant. Eight different lists consisting of 128 stimuli each were created for each language, avoiding the repetition of the same set of pictures between languages.

Each trial included the following elements: a fixation point presented for 750 ms followed by the picture to be named which appeared for up to 2000 ms or until response was provided. After each block, participants were allowed to rest. In order to reduce the number of errors due to possible name disagreement/confusion, participants were presented with the set of pictures before the experimental task and were asked to name them in the required language. Participants were tested in two languages (Catalan and Spanish) and, when possible, over two different sessions staggered week apart. The order of language testing was counterbalanced across participants.

The dependent variables were naming latencies (RTs), which were analyzed off-line with Checkvocal (Protopapas, 2007), and accuracy. Errors were classified as follows: ‘No response,’ when the patient was unable to name the object; ‘semantic,’ when they produced an incorrect word semantically related to the target; ‘cross-language intrusion,’ when they produced the correct word but in the incorrect language; ‘phonological paraphasia,’ when they deleted, substituted or added phonemes to the correct word describing the picture; and ‘unrelated,’ when they produced a word with no relation, semantic or otherwise, to the target word.

#### Bilingual Word-Picture Matching Task

Stimuli were made up of 60 pictures from different semantic categories selected from Snodgrass and Vanderwart (1980) database. A list of 240 words was also selected consisting of two types of stimuli: (a) 120 as target words corresponding to the picture presented (60 in Catalan and 60 in Spanish); (b) 120 as distractor words semantically related to target words (60 in Catalan and 60 in Spanish). Distractor and target words were of the same semantic category. Each picture was presented with a pair of words, one being the target and the other being the distractor. The pictures and the words were presented in a mixed language condition (Catalan and Spanish), but within each trial the two words were from the same language. There were two types of trials: repeat trials in which participants had to match the picture to a target word in the same language as the target of the previous trial, and switch trials in which participants were required to do the matching within the opposite language compared to the previous trial. There were a total of 120 trials presented in the following manner: 43 Spanish repeat trials, 43 Catalan repeat trials, 17 Spanish switch trials and 17 Catalan switch trials; the task was thus comprised of 28% switch trials and 72% repeat trials. Every trial started with a fixation point (a black cross) in the center of the screen displayed for 500 ms, followed by a picture and two words below for a maximum of 2500 ms. Participants were required to match the target word with the picture presented on the screen by pressing one of two keys on the keyboard. The two keys used for the response corresponded to the word appearing on the same side of the computer as the key (i.e., “z” corresponding to the word on the left side of the screen). Dependent variables were defined as RTs and accuracy.

#### Flanker Task

Target stimuli consisted of a row of five horizontal black lines with arrowheads pointing left or right, with the central arrow acting as the true target. Participants were instructed to indicate the direction (left or right) of the central arrow via pressing one of two keys on the keyboard. The target (central arrow) was presented in two main conditions: with congruent flankers (same direction as the target) and incongruent flankers (opposite direction). The event presentation was as follows: (a) a fixation point (a plus sign) appeared at the center of the screen for 400 ms, and (b) the target arrow and the flankers were presented simultaneously until the participants responded or for up to 2000 ms. The experiment consisted of two blocks of 48 trials each, for a total of 96 trials. The proportion of congruent trials was 75% (*n* = 72) to 25% for incongruent trials (*n* = 24). Participants gave their responses by pressing either the ‘V’ or ‘M’ key according to the direction in which the arrow target was pointing. The dependent variables were RTs and accuracy.

## Results

### Language Impairment in Two Languages

For each participant, we compared the scores of the BAT-C of the two languages using a Chi-squared test with Yates’ correction; ten out of eleven patients showed parallel language deficits (only Pt10 showed a significantly more impaired score in their non-dominant compared to their dominant language, see Table 1).

For connected speech, paired *t*-tests were used to analyze differences between languages (dominant vs. non-dominant); and no difference was found between the two languages in any patient [log type/log token: dominant language = 0.87, non-dominant language = 0.86; *t*(10) = −0.09, *p* = 0.92] (see Table 1).

These two results show that our patients had parallel language impairments.

### Semantic Blocked Cyclic Naming Task

We first explored the effects of semantic blocking in healthy individuals by performing repeated-measures ANOVAs including Condition (Homogenous vs. Heterogeneous), Language (Dominant vs. Non-dominant), and Cycle (1, 2, 3, and 4) as within-subject factors in the control group only. In a further analysis, we performed repeated-measures ANOVAs including the same within-subject factors and Group as a between-subject factor (patients with aphasia vs. healthy controls). The analyses were performed for two dependent variables—RTs and accuracy—separately. RTs were analyzed for correct responses only. Moreover, RTs across all conditions exceeding three standard deviations above or below mean were excluded from the analyses for each participant.

#### Reaction Times (RTs)

The analysis with healthy controls showed that main effects of Condition [*F*(1,12) = 1307, *p* = 0.004, $\eta_{p}^{2}$ = 0.52] and Cycle [*F*(3,36) = 17.41, *p* < 0.001, $\eta_{p}^{2}$ = 0.59] were significant, but not Language [*F*(1,12) = 0.05, *p* = 0.82]. The interaction between Condition and Cycle was also significant [*F*(3,36) = 5.79, *p* = 0.002, $\eta_{p}^{2}$ = 0.33]. *Post hoc* analyses showed that, in the Heterogeneous condition, naming latencies became faster over cycle (1st cycle: *M* = 712.91 ms, *SD* = 34.88; 2nd cycle: *M* = 664.67, *SD* = 27.47 ms; 3rd cycle: *M* = 639.18 ms, *SD* = 27.01; 4th cycle: *M* = 629.23 ms, *SD* = 24.06; *p*_s_ < 0.05). On the other hand, naming latencies in Homogeneous conditions only decreased from the first (*M* = 709.32 ms, *SD* = 27.71) to the second cycle (*M* = 672.24 ms, *SD* = 28.72) (*p* = 0.04). No other interaction was significant.

**FIGURE 1:**
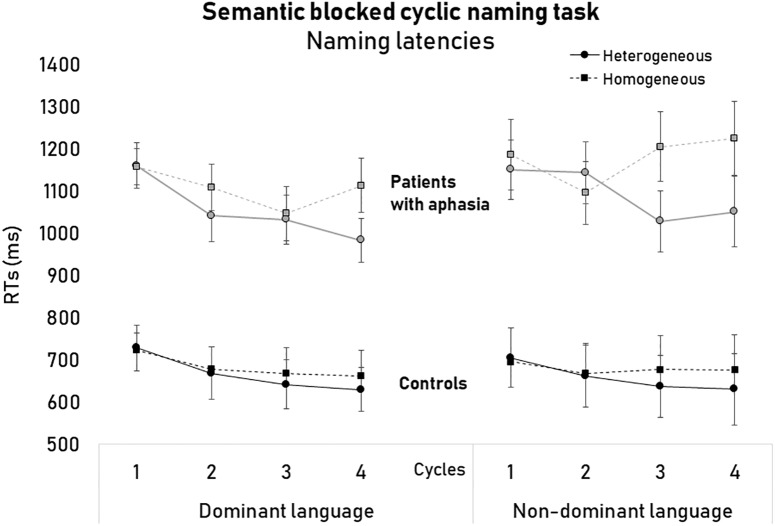
Naming latencies (ms) of the semantic blocked cycling naming task as a function of languages, semantic conditions, cycles, and groups of participants.

The analysis that included both groups showed that the main effects of Condition [*F*(1,22) = 58.12, *p* < 0.001, $\eta_{p}^{2}$ = 0.72] and Cycle [*F*(1,22) = 9.28, *p* < 0.001, $\eta_{p}^{2}$ = 0.29] were significant, but not Language [*F*(1,23) = 0.52, *p* = 0.48]. Also, the main effect of Group was significant [*F*(1,22) = 39.79, *p* < 0.001, $\eta_{p}^{2}$ = 0.64] indicating that patients overall were slower (*M* = 1107.41 ms, *SD* = 44.05) than controls (*M* = 671.75 ms, *SD* = 46.87) in performing the task (see Figure 1 and Table 3).

**Table 3:** RTs in the semantically blocked cyclic naming task for healthy controls and patients with aphasia.

Dominant language								
	*Cycle 1*		*Cycle 2*		*Cycle 3*		*Cycle 4*	
	*M*	*SE*	*M*	*SE*	*M*	*SE*	*M*	*SE*
**Patients with aphasia**								
*Heterogeneous*	1160	54	1041	62	1032	58	983	52
*Homogeneous*	1158	43	1109	54	1046	64	1113	64
**Healthy controls**								
*Heterogeneous*	728	52	668	60	642	55	630	50
*Homogeneous*	723	41	678	52	668	62	662	61
Non-dominant language								
**Patients with aphasia**								
*Heterogeneous*	1151	70	1143	74	1028	73	1051	84
*Homogeneous*	1186	84	1095	74	1205	83	1225	87
**Healthy controls**								
*Heterogeneous*	705	68	661	71	637	70	630	81
*Homogeneous*	695	80	668	71	678	80	676	84

The interaction between Condition and Cycle was also significant [*F*(3,66) = 5.25, *p* = 0.003, $\eta_{p}^{2}$ = 0.19]. *Post hoc* analyses showed that in the Heterogeneous condition naming latencies became faster from the first cycle (*M* = 960.27 ms, *SD* = 33.98) to the second (*M* = 917.93 ms, *SD* = 34.17) to the third cycle (*M* = 873.25 ms, *SD* = 39.12) (*p*_s_ < 0.05). On the other hand, naming latencies in Homogeneous conditions only decreased from the first (*M* = 966.16, *SD* = 32.45 ms) to the second cycle (*M* = 918.51 ms, *SD* = 36.78) (*p* = 0.03).

Finally, the Language × Condition × Cycle interaction [*F*(3,66) = 4.05, *p* = 0.01, $\eta_{p}^{2}$ = 0.15] as well as the Language × Condition × Cycle × Group interaction were significant [*F*(3,69) = 3.15, *p* = 0.03, $\eta_{p}^{2}$ = 0.12]. Further analyses were conducted by comparing the semantic interference effects (difference in naming latencies between the Homogenous and the Heterogeneous condition) within the two groups of participants for each language separately. In the non-dominant language, the magnitude of the semantic interference effect was larger in patients than in controls for the cycles 3 [patients: *M* = 178.27 ms, *SD* = 41.90; controls: *M* = 39.69 ms, *SD* = 38.54; *F*(1,24) = 5.92, *p* = 0.02, $\eta_{p}^{2}$ = 0.21] and 4 [patients: *M* = 182.36 ms, *SD* = 52.61; controls: *M* = 39.46 ms, *SD* = 48.40; *F*(1,24) = 3.99, *p* = 0.05, $\eta_{p}^{2}$ = 0.15]. In the dominant language, the magnitude of the semantic interference effect did not differ between patients and healthy controls across cycles (all *p*s > 0.05).

#### Accuracy

The analysis with healthy controls revealed no main effect or interaction that was statistically significant.

The analysis with both groups showed that the main effect of Group was significant [*F*(1,22) = 14.51, *p* = 0.001, $\eta_{p}^{2}$ = 0.40], indicating that the patients’ performance (*M* = 82.25%, *SD* = 3.22) was lower than that of controls (*M* = 98.83%, *SD* = 2.95). Also, the main effect of Cycle [*F*(3,66) = 7.33, *p* < 0.001, $\eta_{p}^{2}$ = 0.25] and the interaction between Cycle and Group [*F*(3,66) = 5.61, *p* = 0.002, $\eta_{p}^{2}$ = 0.20] were significant. *Post hoc* analyses reveal that patients, but not controls, showed little increase of accuracy in the cycle 3 (*M* = 83.93%, *SD* = 3.09, *p* = 0.03) and 4 (*M* = 84.34%, *SD* = 2.96, *p* = 0.02) compared to the first (*M* = 78.91%, *SD* = 3.62).

#### Error Analysis

The frequency of error types for the two languages is detailed below:

- No response: 54.23% of dominant language errors and 51.10% of non-dominant language errors;
- Phonological errors: 12.31% of dominant language errors and 11.16% of non-dominant language errors;
- Semantic errors: 14.46% of dominant language errors and 15.61% of non-dominant language errors;
- Cross-language intrusions: 9.23% of dominant language errors and 12.26% of non-dominant language errors;
- Unrelated: 9.77% of dominant language errors and 9.87% of non-dominant language errors.

### Bilingual Word-Picture Matching Task

In an initial analysis, repeated-measures ANOVAs were performed including Type of Trial (repeat vs. switch) and Language (Dominant vs. Non-dominant) as within-subject factors in healthy controls only. Following said analysis, repeated-measures ANOVAs were performed with the same within-subject factors but also including Group (patients with aphasia vs. healthy controls) as a between-subject factor. The analyses were performed for two dependent variables—RTs and accuracy—separately. Two patients did not complete this task; therefore, the group comparison was carried out between 10 patients and 13 healthy controls. RTs were analyzed for correct responses only. Moreover, RTs across all conditions exceeding three standard deviations above or below mean were excluded from the analyses for each participant.

#### Reaction Times

The analysis with healthy controls revealed no main effect or interaction that was statistically significant [Type of Trial: *F*(1,12) = 0.87, *p* = 0.37; Language: *F*(1,12) = 0.23, *p* = 0.64; Type of Trial × Language: *F*(1,12) = 0.03, *p* = 0.86].

In the analysis with both groups, the main effect of Type of Trial [*F*(1,22) = 3.28, *p* = 0.08] and Language [*F*(1,22) = 1.57, *p* = 0.22] were not statistically significant. However, the main effect of Group was significant [*F*(1,22) = 57.85, *p* < 0.001, $\eta_{p}^{2}$ = 0.72], indicating that patients (*M* = 1942.52 ms, *SD* = 75.45) performed more slowly than healthy controls (*M* = 1051.88 ms, *SD* = 72.24). Also, the interactions between Type of Trial and Language [*F*(1,22) = 4.95, *p* = 0.04, $\eta_{p}^{2}$ = 0.18] and Type of trial × Language × Group [*F*(1,22) = 4.44, *p* = 0.05, $\eta_{p}^{2}$ = 0.17] were significant (see Table 4).

**Table 4:** RTs and accuracy in the bilingual word-picture matching task for healthy controls and patients with aphasia.

	Healthy controls				Patients with aphasia			
	*Repeat*	*SE*	*Switch*	*SE*	*Repeat*	*SE*	*Switch*	*SE*
**RTs**								
Dominant language	1051	61	1059	82	1949	66	1882	*90*
Non-dominant language	1041	64	1052	87	1938	70	1998	*95*
**Accuracy**								
Dominant language	98.8	1.5	96.5	2.7	94.1	1.6	89.8	2.9
Non-dominant language	98.6	1.1	97.0	2.1	94.1	1.2	92.6	2.2

To explain the triple interaction, further ANOVAs were performed including Type of Trial and Language as within-subject factors for the groups separately. In healthy individuals, no main effect nor interactions were statistically significant [*F*_s_ < 1]. In patients, only the interaction between Type of Trial and Language was significant [*F*(1,10) = 4.87, *p* = 0.05, $\eta_{p}^{2}$ = 0.35]. *Post hoc* analysis showed that patients performed similarly in repeat (*M* = 1949.81, *SD* = 92.91 ms) and switch trials (*M* = 1938.27 ms, *SD* = 128.88 ms; *p* = 0.80) in their dominant language, but significantly slower in switch (*M* = 1998.90 ms, *SD* = 104.61) than repeat (*M* = 1882.09 ms, *SD* = 76.80) trials when they performed the task in their non-dominant language (*p* = 0.04). This result suggests that patients suffered switch cost in their non-dominant language whereas controls did not.

#### Accuracy

In the analysis with healthy controls, we found a main effect of Type of trial to be significant [*F*(1,12) = 7.19, *p* = 0.02, $\eta_{p}^{2}$ = 0.37], suggesting that participants were less accurate in switch (*M* = 96.86%, *SD* = 0.74) than repeat (*M* = 98.82%, *SD* = 0.29) trials (see Table 4). No other main effects or interactions were statistically significant.

In the analysis with both groups, the main effect of Type of trial was significant [*F*(1,22) = 5.11, *p* = 0.03, $\eta_{p}^{2}$ = 0.21], suggesting that participants were less accurate in switch (*M* = 93.91%, *SD* = 1.32) than repeat (*M* = 96.43%, *SD* = 1.53) trials. Also, the main effect of Group was significant [*F*(1,22) = 4.09, *p* = 0.05, $\eta_{p}^{2}$ = 0.17] indicating that patients (*M* = 92.79%, *SD* = 1.71) were less accurate than healthy controls (*M* = 97.84%, *SD* = 1.52). No other main effect or interaction was statistically significant (see Table 4).

### Flanker Task

Repeated-measures ANOVAs were performed including Type of Trial (congruent vs. incongruent) as a within-subject factor and Group (patients with aphasia vs. healthy controls) as a between-subject factor for RTs and accuracy separately. RTs were analyzed for correct responses only. Moreover, RTs across all conditions exceeding three standard deviations above or below mean were excluded from the analyses for each participant.

#### Reaction Times

The main effect of Type of Trial was significant [*F*(1, 22) = 1191.73, *p* < 0.001, $\eta_{p}^{2}$ = 0.85], suggesting than participants were slower in incongruent (*M* = 990.33 ms, *SD* = 285.6) than in congruent (*M* = 879.64 ms, *SD* = 278.52) trials. Also, the main effect of group was significant [*F*(1,22) = 28.31, *p* < 0.001, $\eta_{p}^{2}$ = 0.57], indicating that patients with aphasia were slower (*M* = 1148.32 ms, *SD* = 285.74) than healthy controls (*M* = 711.09 ms, *SD* = 86.19) to perform the task. Finally, the interaction between Type of Trial and Group was not statistically significant [*F*(1,22) = 2.11, *p* = 0.17], suggesting that the magnitude of the conflict cost was the same for the groups (see Table 5).

**Table 5:** RTs and accuracy in the flanker task for healthy controls and patients with aphasia.

	Healthy controls		Patients with aphasia	
Type of trial	*Means*	*SE*	*Means*	*SE*
**RTs**				
Congruent	649	47	1087	51
Incongruent	772	45	1200	49
*Conflict costs*	*123*		*113*	
**Accuracy**				
Congruent	99.9	0.7	98.0	0.6
Incongruent	98.6	1.2	96.5	1.2

#### Accuracy

The main effect of Type of trial was significant [*F*(1,22) = 7.05, *p* = 0.01, $\eta_{p}^{2}$ = 0.25], suggesting higher accuracy in congruent (*M* = 98.97%, *SD* = 4.14) than in incongruent trials (*M* = 97.56%, *SD* = 2.31). However, no significant difference was found between patients with aphasia (*M* = 97.12%, *SD* = 3.07) and healthy controls (*M* = 99.26%, *SD* = 1.45) [*F*(1, 22) = 2.78, *p* = 0.11].

### Correlations Between Linguistic and Non-linguistic Measures

To address one of our hypotheses that language deficits might be related to non-linguistic control deficits, we performed correlations between each individual’s performance on the tasks used to assess both domains.

For the non-linguistic domain, we used the individual speed of processing (congruent and incongruent trials) and the magnitude of the conflict cost (RTs on incongruent trials minus RTs on congruent trials) on the flanker task. For the linguistic domain, we used the magnitude of the semantic interference effect (RTs in homogeneous blocks minus RTs in heterogeneous block) and switch costs at the individual level for both dominant and non-dominant languages within the semantic blocked cyclic naming task and the bilingual matching task, respectively.

**FIGURE 2:**
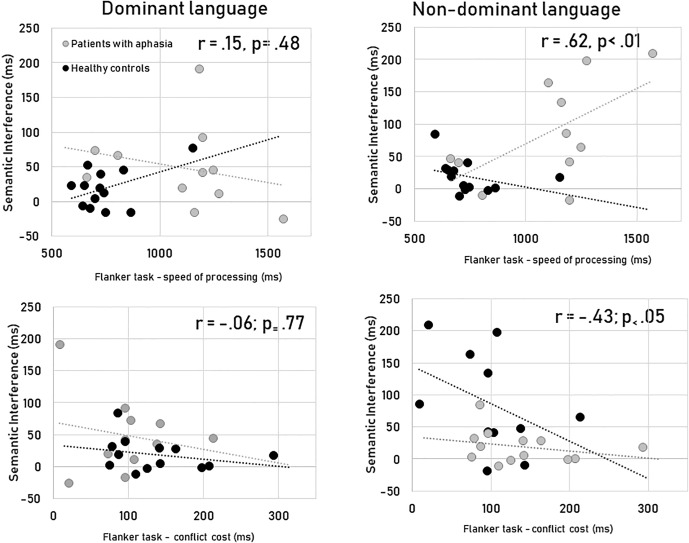
Correlations between non-linguistic (speed of processing and conflict cost) and the linguistic (semantic interference) measures for the two languages.

For the dominant language, the magnitude of the semantic interference did not correlate with the speed of processing [*r*(24) = 0.15, *p* = 0.48] and the conflict cost of the flanker task [*r*(24) = −0.06; *p* = 0.77] (see Figure 2). The switch costs in their dominant language did correlate with the speed of processing [*r*(24) = 0.89, *p* < 0.001], but not with the cost seen in the flanker task [*r*(24) = −0.13; *p* = 0.55].

However, for the non-dominant language, the magnitude of the semantic interference did correlate with the speed of processing [*r*(24) = 0.62, *p* = 0.001] and the conflict cost of the flanker task [*r*(24) = −0.43; *p* = 0.05]. The switch costs for the non-dominant language correlated with the speed of processing [*r*(24) = 0.86, *p* < 0.001] but not with the cost seen in the flanker task [*r*(24) = −0.26; *p* = 0.22].

Moreover, the degree of language impairment as indexed by the AQ of the WAB did not correlate with either non-linguistic [speed of processing: *r*(10) = −0.35, *p* = 0.16; conflict cost: *r*(10) = 0.19, *p* = 0.61] or linguistic performance in patients for both languages on the semantic blocked naming task [dominant language: *r*(10) = −0.45, *p* = 0.14; non-dominant language: *r*(10) = 0.41, *p* = 0.24] and on the bilingual matching task [dominant language: *r*(10) = 0.09, *p* = 0.82; non-dominant language: *r*(10) = 0.15, *p* = 0.70].

## Discussion

With this study we aimed to investigate the language dependency of semantic processing in bilinguals. To address this question, we explored the performance of bilinguals with fluent aphasia and parallel language impairment on tasks of production and comprehension within their two languages. Furthermore, we used an EC task to explore whether the control mechanisms in the linguistic and non-linguistic domain may overlap.

We found three main results. First, semantic control processes related to lexical selection are language-dependent, as measured by a larger semantic interference effect during non-dominant language production in bilingual patients with aphasia. Second, the retrieval of semantic representations might have also a certain degree of language dependency under specific conditions, such as dual language contexts. Third, the linguistic processes of semantic control show only partial overlap with those of domain-general EC (i.e., during conflict monitoring).

### Language Dependency of Semantic Control in Production

We found evidence of language dependency for the semantic control system in bilinguals. Our bilingual patients with aphasia showed a higher semantic interference effect than healthy controls and, interestingly, to a greater degree when they did the task in their non-dominant language. First, it is important to stress that this result cannot be attributed to an imbalance of proficiency in the two languages in patients. In studies such as ours, it becomes necessary to exclude this variable as one of the factors that could explain differences in semantic processing between languages in bilingual patients with aphasia (Kiran and Edmonds, 2004; Lorenzen and Murray, 2008; Kiran and Roberts, 2010; Kiran et al., 2013a,b; Khachatryan et al., 2016). Given that patients in our study acquired their non-dominant language early on and had a similar frequency of usage in both their languages before injury, this possible confounding factor of language proficiency cannot account for the greater semantic interference in the non-dominant language observed.

Our results regarding semantic control complement previous studies that investigated the network of semantic representation in bilinguals. As reviewed in the Introduction, most of the neuroimaging studies have shown that bilinguals use very similar neural networks while they process semantic features of their L1 and L2 (e. g., Illes et al., 1999; Chee et al., 2001; Pillai et al., 2003; Grogan et al., 2009; Correia et al., 2014; Van de Putte et al., 2017). Similarly, neuropsychological studies of bilingual patients with semantic memory impairment indicate a comparable decline of the two languages, suggesting a common and shared neural network in the temporal lobe (Mendez et al., 1999; Hernández et al., 2008, 2010). However, it is important to highlight that, in the case of bilingual aphasia, we do not expect a deficit in semantic memory at the representational level, but rather a deficit in the control components of semantic retrieval (Jefferies and Lambon Ralph, 2006; Noonan et al., 2010). Similar to some extent to the previous concept of “access,” semantic control is in charge of retrieving the semantic information needed for a specific context and depends upon cognitive demand (Jefferies et al., 2008). Given this distinction, semantic control would be more affected in patients having lesions in fronto-temporoparietal areas due to decreased capacity to inhibit semantic competitors while their semantic representations could be spared (Jefferies and Lambon Ralph, 2006; Jefferies et al., 2008; Rogers et al., 2015). Therefore, we believe that our patients relied more on these control processes, within the linguistic domain, while they named pictures in those semantically blocked conditions where they were required to inhibit competitors. This type of competitive process has consequences at the lexical level, during selection and retrieval of the words. Accordingly, although we manipulated the degree of semantic competition within our task, we cannot exclude a possible effect at the lexical level since it is interconnected with the semantic units.

Different hypotheses have been proposed for the pathological effects found in patients when they have to name elements within semantically homogeneous conditions (Schnur et al., 2006, 2009; Harvey and Schnur, 2015). Our results seem to suggest that the problem experienced by aphasic patients in reducing semantic competition possibly comes from an excessive inhibition of lexical representations (McCarthy and Kartsounis, 2000). The semantic similarity between items would cause an increased level of inhibition on non-target words that would spread throughout the network; this same inhibition would then make a following, semantically related lexical item less accessible. Indeed, patients with aphasia showed more omissions than semantic errors, supporting the notion that they were not able to retrieve the correct name because it was completely inhibited. Moreover, this inhibitory process seems to be within-language since our patients did not produce many cross-language intrusions. This interpretation is more compatible with our findings than other hypotheses that proposed over-activation at the semantic level that builds up across cycles (Belke et al., 2005; Schnur et al., 2006; for a non-competitive selection account see Navarrete et al., 2014).

Interestingly, patients’ ability to inhibit competitors during lexical retrieval was especially reduced while they were performing the task in their non-dominant language. This is not to say that the semantic representations of their non-dominant language were more affected. Rather, in control-demanding situations such as naming in their non-dominant language during homogenous conditions, lexical retrieval engaged the control network of semantic cognition to a greater degree and, in turn, resulted in a slowing down of the process. These results could be explained by some of the models of bilingual language production that have proposed language-dependency of lexico-semantic processing. For instance, Kroll and Stewart (1994) asserted that the lexico-semantic connections between L2 and L1 are weaker than those between L1 and L2; Gollan et al. (2008) also claimed that difference in frequency of language usage might explain why L2 retrieval is more demanding for bilinguals. However, Kroll and Stewart (1994)’s proposal is mainly based on data with late bilinguals and the predictions of their model are not entirely applicable to the population of early bilinguals that we studied (for a critical discussion of this issue, see Hernández et al., 2010). The only way to interpret our results with the Kroll and Stewart (1994)’s model would be to assume difference in the level of activation for lexical competition of the two languages (Kroll et al., 2010).

### Language Dependency of Semantic Control in Comprehension

Differential language impairment observed in speech production also extended to comprehension. Our main aim was originally to study semantic processing during word production, but we decided to also include a comprehension task to check the integrity of the semantic representations. Although the matching task was not designed to measure the blocking and cycling effects of semantic interference, we found that when the two languages are mixed in a semantic matching task, the non-dominant is more affected than the dominant one. Conversely, healthy controls did not show any switch cost and this is probably due to the nature of task. The studies that have used semantic categorization and lexical decision in healthy individuals have showed that switch costs are not always reliable in the matching tasks or they are reduced compared to production, probably because they require less involvement of language activation and inhibition (e.g., Orfanidou and Sumner, 2005; Macizo et al., 2012; Mosca and de Bot, 2017). We might say that this result in patients at the comprehension level could be related to some deficits in the access of lexical representations for the non-dominant language. Following brain damage, the competitive process (possibly of inhibitory nature) for lexical selection in the non-dominant language could be affected and this would explain why patients are more impaired in that language. Previous neuroimaging studies in bilinguals found mixed results: some that the control network described for language production (Abutalebi and Green, 2007, 2016; Calabria et al., 2018) is also active during word comprehension and recognition tasks (Peeters et al., 2019), but some other studies suggest that the overlap between the two system is only partial (Abutalebi et al., 2007; Blanco-Elorrieta and Pylkkänen, 2017).

These results show that the language-dependent nature of semantic control processes in bilinguals with aphasia during word production in single language contexts extends to word comprehension in dual language contexts. However, caution is required when interpreting these results due to important methodological differences between the two tasks. Despite the fact that the bilingual word-picture matching task could also be defined as a semantic task, participants performed it in a dual language condition, whereas, in the semantic blocked naming task, the two languages were not mixed. Future research should examine whether semantic control processes continue to exhibit a language-dependent nature during word comprehension when tested in single language contexts.

### Semantic Control and EC in Bilinguals

The ‘executive selection account’ proposes that the effect of interference generated in the semantic blocked cyclic naming task is mediated by the involvement of domain-general EC mechanisms that are outside of the linguistic domain (Wilshire and McCarthy, 2002). In fact, there is evidence that performance on the Stroop task correlates with naming latencies in homogeneous conditions, suggesting that inhibition at response selection level would be the same in EC and semantic control (Crowther and Martin, 2014). Similarly, the involvement of the left inferior frontal gyrus and the left caudate nucleus has been interpreted as the EC network being responsible for resolving interference of semantic competitors (Canini et al., 2016). Given this evidence, we included the flanker task to measure individual domain-general EC performance.

Our results partially support this account. We found a positive correlation between the magnitude of the semantic interference effect and the speed of processing in the flanker task, but only for the non-dominant language. There was also a negative correlation between semantic interference and conflict cost, suggesting that a reduced magnitude of semantic interference is associated with smaller conflict costs. This observation is likely biased by patients’ performance: given they are already very slow to respond in the congruent conditions, their “reduction” in conflict cost might be reflecting this generalized slowness rather than a true decrease in cost. In any case, this result seems to indicate that semantic competition does not overlap with general-domain EC mechanisms responsible for conflict resolution, contrary to what other studies have suggested (Crowther and Martin, 2014; Canini et al., 2016). Moreover, our patients were not impaired in conflict resolution as they had similar levels of conflict costs as healthy controls. However, they were generally slower, suggesting that the EC deficit they likely possessed was in conflict monitoring (Botvinick et al., 1999; Botvinick et al., 2001; Yeung, 2013). Conflict monitoring allows for the detection of potentially conflicting situations and subsequent adjustment of behavior when there is a switch from non-conflict situations (congruent trials) to conflict ones (incongruent trials) and vice versa. Therefore, the positive correlation between semantic interference and speed of processing may be interpreted in terms of an overlap of monitoring abilities (or deficits in these abilities for aphasic patients) between the linguistic domain (semantic control) and non-linguistic EC. Conflict resolution has been related to frontal activity (anterior cingulate cortex, Botvinick et al., 2001; for evidence in bilinguals see Abutalebi et al., 2012) and it is possible that this region was spared in our patients since they have fluent aphasia, a type of language disorder more strongly related to temporo-parietal damage. Therefore, we have to acknowledge the possibility that they did not show a deficit in conflict resolution because they did not have brain damage in frontal areas. Indeed, we know that higher semantic interference effects in bilingual patients are related to EC deficits when they have brain damage extending to the inferior frontal gyrus, as shown in a study by Schnur et al. (2009).

Previous studies that have compared linguistic to non-linguistic performance using a flanker task in bilingual patients with aphasia (Green et al., 2010; Verreyt et al., 2013; Dash and Kar, 2014; Gray and Kiran, 2015) have shown that there is an incomplete overlap between the two control systems. Further studies should explore other EC components, such as working memory and switching abilities, to determine whether these non-linguistic control mechanisms are more closely related to language control deficits in bilingual speakers.

## Conclusion

The results of our study suggest that semantic control may be language-dependent and selective language impairment could be explained by an excessive inhibition placed upon the lexical representations of the non-dominant language. Additionally, semantic interference seems to be at least somewhat related to conflict monitoring deficits, suggesting a certain degree of overlap between EC and semantic control.

## Ethics Statement

This study was carried out in accordance with the recommendations of the “Parc de Salut MAR - Research Ethics Committee” with written informed consent from all subjects. All subjects gave written informed consent in accordance with the Declaration of Helsinki. The protocol was approved by the “Parc de Salut MAR - Research Ethics Committee” (reference number: 2018/8029/I).

## Author Contributions

MC conceived the research, designed the experiments, and analyzed the data. NG, CS, and MS collected the experimental and neuropsychological data. Moreover, all the authors equally contributed to writing of the manuscript and discussion of the results.

## Conflict of Interest Statement

The authors declare that the research was conducted in the absence of any commercial or financial relationships that could be construed as a potential conflict of interest.
