# Supplementary material for: Semantic Processing in Bilingual Aphasia: Evidence of Language Dependency
Source: Front Hum Neurosci. 2019 Jun 14;13:205. doi: 10.3389/fnhum.2019.00205 (PMC6587373; doi:10.3389/fnhum.2019.00205)
Supplement: Supplementary file 1 [file Data_Sheet_1.docx]

**Appendix I** – Individual data of the socio-demographic and linguistic characteristics of the sample

|  |  |  |  |  |  |  |  |  | **Dominant language (DL) proficiency** | | | | **Non-dominant language (NDL) proficiency** | | | |  |
| --- | --- | --- | --- | --- | --- | --- | --- | --- | --- | --- | --- | --- | --- | --- | --- | --- | --- |
| **Participant** | **Age** | **Education (years)** | **Gender** | **Dominant** |  | **Non-Dominant** | **Age of regular use (DL)** | **Age of regular use (NDL)** | **Speaking** | **Comprehension** | **Reading** | **Writing** | **Speaking** | **Comprehension** | **Reading** | **Writing** | **% Language usage** |
| Pt1 | 52 | 12 | M | CAST |  | CAT | 2 | 7 | 4 | 4 | 4 | 3 | 3 | 3 | 3 | 3 | 40 |
| Pt2 | 45 | 15 | M | CAT |  | CAST | 2 | 5 | 4 | 4 | 4 | 4 | 4 | 4 | 4 | 4 | 68 |
| Pt3 | 54 | 12 | M | CAT |  | CAST | 2 | 4 | 4 | 4 | 4 | 4 | 4 | 4 | 4 | 4 | 65 |
| Pt4 | 57 | 12 | M | CAT |  | CAST | 2 | 2 | 4 | 4 | 3 | 3 | 4 | 4 | 4 | 4 | 58 |
| Pt5 | 44 | 12 | H | CAT |  | CAST | 3 | 3 | 4 | 4 | 4 | 4 | 4 | 4 | 4 | 4 | 53 |
| Pt6 | 64 | 16 | M | CAST |  | CAT | 2 | 6 | 4 | 4 | 4 | 4 | 4 | 4 | 4 | 4 | 33 |
| Pt7 | 67 | 15 | H | CAT |  | CAST | 2 | 5 | 4 | 4 | 4 | 4 | 4 | 4 | 4 | 4 | 48 |
| Pt8 | 56 | 15 | H | CAT |  | CAST | 2 | 5 | 4 | 4 | 4 | 4 | 4 | 4 | 4 | 4 | 52 |
| Pt9 | 61 | 15 | H | CAT |  | CAST | 2 | 2 | 4 | 4 | 3 | 3 | 4 | 4 | 4 | 4 | 69 |
| Pt10 | 68 | 12 | H | CAST |  | CAT | 2 | 5 | 4 | 4 | 4 | 4 | 4 | 4 | 4 | 4 | 45 |
| Pt11 | 65 | 15 | M | CAT |  | CAST | 2 | 6 | 4 | 4 | 3 | 3 | 4 | 4 | 4 | 4 | 72 |
| Control1 | 63 | 15 | M | CAT |  | CAST | 2 | 6 | 4 | 4 | 4 | 4 | 4 | 4 | 4 | 4 | 67 |
| Control2 | 53 | 15 | M | CAT |  | CAST | 2 | 5 | 4 | 4 | 4 | 4 | 4 | 4 | 4 | 4 | 72 |
| Control3 | 53 | 15 | M | CAT |  | CAST | 2 | 4 | 4 | 4 | 4 | 4 | 4 | 4 | 4 | 4 | 32 |
| Control4 | 53 | 15 | M | CAT |  | CAST | 2 | 4 | 4 | 4 | 4 | 4 | 4 | 4 | 4 | 4 | 58 |
| Control5 | 52 | 13 | M | CAT |  | CAST | 3 | 6 | 4 | 4 | 4 | 4 | 4 | 4 | 4 | 4 | 48 |
| Control6 | 53 | 15 | M | CAT |  | CAST | 2 | 5 | 4 | 4 | 4 | 4 | 4 | 4 | 4 | 4 | 50 |
| Control7 | 61 | 15 | M | CAT |  | CAST | 2 | 3 | 4 | 4 | 4 | 4 | 4 | 4 | 4 | 4 | 70 |
| Control8 | 52 | 15 | M | CAT |  | CAST | 3 | 3 | 4 | 4 | 4 | 4 | 4 | 4 | 4 | 4 | 45 |
| Control9 | 62 | 12 | M | CAT |  | CAST | 2 | 5 | 4 | 4 | 4 | 4 | 4 | 4 | 4 | 4 | 56 |
| Control10 | 57 | 12 | H | CAST |  | CAT | 2 | 6 | 4 | 4 | 4 | 4 | 4 | 4 | 3 | 4 | 28 |
| Control11 | 54 | 15 | H | CAST |  | CAT | 2 | 8 | 3 | 4 | 4 | 3 | 4 | 4 | 4 | 4 | 5 |
| Control12 | 55 | 15 | H | CAST |  | CAT | 2 | 6 | 4 | 4 | 4 | 4 | 4 | 4 | 4 | 4 | 14 |
| Control13 | 52 | 15 | M | CAT |  | CAST | 3 | 6 | 4 | 4 | 4 | 4 | 4 | 4 | 4 | 4 | 41 |

**Appendix II** – List of stimuli

| **Spanish name** | **Catalan name** | **English name** | **Cognate status** | **Frequency Spanish** | **Frequency Catalan** | **Word length Spanish (syllables)** | **Word length Catalan (syllables)** |
| --- | --- | --- | --- | --- | --- | --- | --- |
| ***Vegetables*** |  |  |  |  |  |  |  |
| lechuga | enciam | salad | Non-cognate | 2.86 | 544 | 3 | 2 |
| zanahoria | pastanaga | carrot | Non-cognate | 2.32 | 314 | 4 | 4 |
| pimiento | pebrot | pepper | Non-cognate | 1.61 | 335 | 3 | 2 |
| seta | bolet | mushroom | Non-cognate | 0.34 | 1074 | 2 | 2 |
| cebolla | ceba | onion | Cognate | 9.46 | 1152 | 3 | 2 |
| tomate | tomàquet | tomato | Cognate | 6.79 | 811 | 3 | 3 |
| calabaza | carabassa | pumpkin | Cognate | 2.5 | 529 | 4 | 4 |
| espárrago | espàrrec | asparagus | Cognate | 2.32 | 239 | 4 | 3 |
| ***Animals*** |  |  |  |  |  |  |  |
| conejo | conill | rabbit | Non-cognate | 6.61 | 1668 | 3 | 2 |
| murciélago | ratpenat | bat | Non-cognate | 3.04 | 257 | 4 | 3 |
| mariposa | papallona | butterfly | Non-cognate | 6.25 | 1024 | 4 | 4 |
| pato | ànec | duck | Non-cognate | 4.82 | 528 | 2 | 2 |
| rana | granota | frog | Non-cognate | 6.25 | 762 | 2 | 3 |
| búho | mussol | owl | Non-cognate | 4.46 | 414 | 2 | 2 |
| cerdo | porc | pig | Non-cognate | 13.93 | 1752 | 2 | 1 |
| gusano | cuc | worm | Non-cognate | 4.29 | 1079 | 4 | 1 |
| ***Kitchen tools*** |  |  |  |  |  |  |  |
| sarten | paella | pan | Non-cognate | 3.21 | 543 | 2 | 3 |
| tenedor | forquilla | fork | Non-cognate | 3.75 | 297 | 3 | 3 |
| cuchillo | ganivet | knife | Non-cognate | 15.36 | 1472 | 3 | 3 |
| vaso | got | glass | Non-cognate | 37.82 | 1048 | 2 | 1 |
| plato | plat | plate | Cognate | 30.89 | 3632 | 2 | 1 |
| cenicero | cendrer | ashtray | Cognate | 6.43 | 106 | 4 | 2 |
| jarra | gerra | pitcher | Cognate | 3.93 | 679 | 2 | 2 |
| botella | ampolla | bottle | Non-cognate | 31.61 | 1837 | 3 | 3 |
| ***Furniture*** |  |  |  |  |  |  |  |
| banco | banc | bank | Cognate | 46.96 | 3737 | 2 | 1 |
| ventana | finestra | window | Non-cognate | 93.93 | 5975 | 3 | 3 |
| armario | armari | closet | Cognate | 21.43 | 1213 | 3 | 3 |
| mesa | taula | table | Non-cognate | 172.14 | 12587 | 2 | 2 |
| cómoda | calaixera | commode | Non-cognate | 10.71 | 466 | 3 | 3 |
| cuna | bressol | crib | Non-cognate | 12.32 | 781 | 2 | 2 |
| silla | cadira | chair | Non-cognate | 48.21 | 4095 | 2 | 3 |
| cama | llit | bed | Non-cognate | 136.43 | 8671 | 2 | 1 |
